# l-Arabinose triggers its own uptake via induction of the arabinose-specific Gal2p transporter in an industrial *Saccharomyces cerevisiae* strain

**DOI:** 10.1186/s13068-018-1231-8

**Published:** 2018-08-23

**Authors:** Verena Oehling, Paul Klaassen, Oliver Frick, Christian Dusny, Andreas Schmid

**Affiliations:** 1 Laboratory of Chemical Biotechnology, Department of Biochemical & Chemical Engineering, TU Dortmund University, Dortmund, Germany; 2 DSM Biotechnology Center, Delft, The Netherlands; 3 Present Address: Department of Solar Materials, Helmholtz Centre for Environmental Research UFZ, Leipzig, Germany

**Keywords:** l-Arabinose uptake, Lignocellulosic ethanol, Gal2p, *Saccharomyces cerevisiae*, Catabolite repression, l-Arabinose fermentation, Next generation sequencing, RNA-seq

## Abstract

Bioethanol production processes with *Saccharomyces cerevisiae* using lignocellulosic biomass as feedstock are challenged by the simultaneous utilization of pentose and hexose sugars from biomass hydrolysates. The pentose uptake into the cell represents a crucial role for the efficiency of the process. The focus of the here presented study was to understand the uptake and conversion of the pentose l-arabinose in *S. cerevisiae* and reveal its regulation by d-glucose and d-galactose. Gal2p—the most prominent transporter enabling l-arabinose uptake in *S. cerevisiae* wild-type strains—has an affinity for the transport of l-arabinose, d-glucose, and d-galactose. d-Galactose was reported for being mandatory for inducing *GAL2* expression. *GAL2* expression is also known to be regulated by d-glucose-mediated carbon catabolite repression, as well as catabolite inactivation. The results of the present study demonstrate that l-arabinose can be used as sole carbon and energy source by the recombinant industrial strain *S. cerevisiae* DS61180. RT-qPCR and RNA-Seq experiments confirmed that l-arabinose can trigger its own uptake via the induction of *GAL2* expression. Expression levels of *GAL2* during growth on l-arabinose reached up to 21% of those obtained with d-galactose as sole carbon and energy source. l-Arabinose-induced *GAL2* expression was also subject to catabolite repression by d-glucose. Kinetic investigations of substrate uptake, biomass, and product formation during growth on a mixture of d-glucose/l-arabinose revealed impairment of growth and ethanol production from l-arabinose upon d-glucose depletion. The presence of d-glucose is thus preventing the fermentation of l-arabinose in *S. cerevisiae* DS61180. Comparative transcriptome studies including the wild-type and a precursor strain delivered hints for an increased demand in ATP production and cofactor regeneration during growth of *S. cerevisiae* DS61180 on l-arabinose. Our results thus emphasize that cofactor and energy metabolism demand attention if the combined conversion of hexose and pentose sugars is intended, for example in biorefineries using lignocellulosics.

**Electronic supplementary material:**

The online version of this article (10.1186/s13068-018-1231-8) contains supplementary material, which is available to authorized users.

## Background

The limited availability of crude oil and the increasing need for the reduction of greenhouse gases has resulted in an increased demand for second generation biofuels as more environmentally friendly energy carriers [1–4]. Second generation bioethanol is commonly produced via fermentation of lignocellulosic biomass hydrolysates with the yeast *Saccharomyces cerevisiae*. In contrast to feedstocks used in first generation ethanol production plants, such as hexose sugars, lignocellulosic biomass consists of the hexose sugars d-glucose, d-mannose, d-galactose, as well as the pentose sugars d-xylose and l-arabinose [5]. Lignocellulosic biomass requires a complex pretreatment (mechanical, chemical) and an enzymatic hydrolysis to liberate biomass-bound sugars and make them accessible for microbial fermentations [3, 6]. The composition of sugar monomers in the hydrolysate varies with the raw material used [7]. d-Xylose is the pentose sugar with the highest abundance in the biomass hydrolysate; it accounts for up to 30% [8–11]. In contrast, l-arabinose has an abundance there of 1.5–2.75% [7, 8]. Only the simultaneous fermentation of sugars contained in the lignocellulosic hydrolysate will lead to cost-efficient and economically feasible ethanol production [12]. For the handling of mixed sugar fermentations and the development of flexible production strains, it is thus important to understand how the single components of the system affect the physiology of the fermenting biocatalyst. This holds especially true for studying the effects of the respective sugars types and its combinations on the productivity of whole-cell biocatalysts with a complex substrate-dependent metabolism like *S. cerevisiae*, which is regulated by the availability of carbon sources via, e.g., carbon catabolite repression [13]. An insufficient understanding of the genetic background can impair strain improvement [14].

**Fig. 1:**
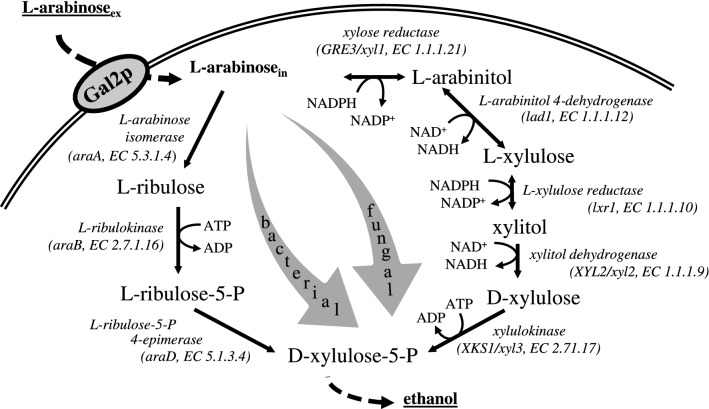
Simplified bacterial (left) and fungal (right) l-arabinose degradation pathway used for metabolic engineering of *S. cerevisiae*. l-Arabinose is taken up via Gal2p in both pathways. The further l-arabinose catabolism is linked to the central carbon metabolism via d-xylulose-5-P, an intermediate of the pentose phosphate pathway. In the fungal route, l-arabinose is reduced to l-arabinitol by the xylose reductase Xyl1p and further oxidized to l-xylulose catalyzed by the l-arabinitol 4-dehydrogenase Lad1p. l-Xylulose is then reduced to xylitol by the l-xylulose reductase Xyl2p, which is further oxidized to d-xylulose by the xylitol dehydrogenase Xyl2p. The conversion of d-xylulose to d-xylulose-5-P by the xylulokinase Xks1p is the final step. In the bacterial route, l-arabinose is converted to l-ribulose by the isomerase *AraA*, phosphorylated to l-ribulose-5-P via the l-ribulokinase *AraB* and converted to d-xylulose-5-P by the l-ribulose-5-P 4-epimerase *AraD*. All steps of the bacterial route are cofactor-independent

The fermentation of hexose sugars is a natural characteristic of *S. cerevisiae* [7, 15, 16]. Large-scale fermentations of the hexose sugars d-glucose, d-mannose, and d-galactose with *S. cerevisiae* are thus established and well-studied processes [17]. However, natural conversion routes for pentoses are lacking in *S. cerevisiae* [18–20]. The presence of pentose sugars in the biomass hydrolysate complicates the fermentation as the introduction of heterologous pathways is necessary [21–24]. Two decades of research and development were required to implement foreign utilization pathways of the pentoses d-xylose and l-arabinose in recombinant *S. cerevisiae*. First d-xylose fermentations with *S. cerevisiae* were reported by Walfridsson et al. in 1996 [25], while the first recombinant l-arabinose pathway in *S. cerevisiae* was reported by the Richard group in 2003 [26]. Since then, pentose pathways for d-xylose and l-arabinose of bacteria as well as of non-*Saccharomyces* yeasts were successfully established in *S. cerevisiae* [12, 18, 23, 27–33] (for l-arabinose see Fig. 1).

The efficiency of ethanol production from d-xylose could be remarkably improved to ethanol yields from d-xylose (up to 0.48 g/g) for recombinant *S. cerevisiae* that account for up to 94% of the maximal theoretical maximum (0.51 g/g) [11, 34]. Hence, the potential of ethanol fermentation from d-xylose during single sugar cultivation is nearly exhausted. Also the co-consumption ratio of d-glucose and d-xylose could be more than doubled by using laboratory evolution and whole genome resequencing [35]. However, constraints in the pentose uptake and in the redox and energy metabolism were identified to still limit ethanol production from l-arabinose [16, 36].

With regard to the energy and redox metabolism, the first four conversion steps of the fungal l-arabinose degradation pathways are redox reactions in which NAD^+^/NADH are involved in oxidation and NADP^+^/NADPH in the reduction reactions of metabolites (see Fig. 1). The resulting redox-imbalance poses a challenge for the application of this pathway as yeast are lacking transhydrogenase activity [7, 27]. By using the redox-independent bacterial l-arabinose degradation pathway, the drawbacks caused by the fungal counterpart could be circumvented [18, 37]. After three enzymatic conversion steps, the formed d-xylulose-5-P is then also channeled into the pentose phosphate pathway (PPP) (see Fig. 1). However, the bacterial l-arabinose utilization pathway causes an additional demand in energy in form of ATP that has to be compensated by the remaining metabolism. The major breakthrough in l-arabinose utilization via the bacterial l-arabinose degradation pathway was reported by Becker and Boles in 2003 [23]. In their approach, *araA* from *Bacillus subtilis* and *araB* and *araD* from *Escherichia coli* were heterologously expressed in *S. cerevisiae.* Additional overexpression of the transporter *GAL2* and cultivation of more than 200 h resulted in a transformant strain that produced ethanol at a specific rate of 0.08 g/(g_cells_ h) [23]. More recently, the plasmid-based introduction of *araABD* from *Lactobacillus plantarum*, the overexpression of the non-oxidative PPP genes and an evolutionary engineering on l-arabinose even yielded a laboratory strain able to consume l-arabinose at rates of 0.7 g/(g_cells_ h) and to produce ethanol at rates of 0.29 g/(g_cells_ h) under complete anaerobic cultivation conditions [28]. These results were obtained by lab-scale investigations of laboratory strains that were cultivated under anaerobic conditions. This however limits the transferability of the obtained knowledge with regard to industrial application due to the following reasons.

First, for commercial applications, it is important to implement pentose-fermentation with industrial strains. Industrial *S. cerevisiae* strains, in comparison to laboratory strains, were found to have an increased robustness against harsh process conditions [38, 39]. Plasmid-based mutants of laboratory strains furthermore bear the risk of plasmid-loss. This requires the uneconomical application of selection markers [40–42]. Several of the currently used industrial strains for ethanol production evolved and optimized their fermentation characteristics during decades of reuse in repeated fed-batch processes [39]. Originally, some of them were even wild yeasts that were captured as a contamination from the used feedstocks [43, 44]. Such natural evolution was based on undirected, randomly occurring mutations and served as model for adaptive evolutionary engineering to improve pentose utilization. Adaptive evolutionary engineering exploits the phenotypic diversity of microbial populations via selecting important phenotypes based on the readout growth [14]. However, the physiology and often more complex genetic architecture of the evolved industrial strains are not as thoroughly characterized as of laboratory strains [14, 17, 37, 39, 44, 45]. To overcome this deficit, we used an industrial cell lineage with the final strain *S. cerevisiae* DS61180 for our investigations regarding l-arabinose utilization. This strain has been constructed by DSM (Delft, NL) via a combination of metabolic and adaptive evolutionary engineering where all changes were stably integrated into the genome [46]. This resulted in a plasmid-free, robust, industrial strain with a stable genome-integrated heterologous l-arabinose degradation pathway. The subsequent adaptive evolutionary engineering towards *S. cerevisiae* DS61180 was exclusively performed under aerobic conditions with a focus on efficient l-arabinose utilization. The strain was thus not capable of growing on d-xylose and was not adapted to use l-arabinose as sole carbon source under anaerobic conditions. For further metabolic engineering strategies for developing robust industrial strains for fermentation of lignocellulosic hydrolysates please refer to Jansen et al. [47].

Second, oxygen availability controls the switch from respiration to fermentation and thus affects physiology, including ethanol and metabolite production, of *S. cerevisiae* [48]. *S. cerevisiae* is one of the very few types of yeast capable of growing under anaerobic conditions [49]. *S. cerevisiae* is a facultative anaerobic microorganism with a respiro-fermentative metabolism [49–51]. Under anaerobic conditions, the fluxes from pyruvate to the TCA cycle are redirected to acetaldehyde, which is converted to ethanol and acetic acid to keep the redox-pool balanced [52]. Without the redirection of metabolic fluxes, redox equivalents from catabolism would accumulate in their reduced form (NADH) as a transhydrogenase that transforms NADH to NADPH is lacking in *S. cerevisiae* [53, 54]. Furthermore, the respiratory chain is inactive when oxygen is unavailable as electron acceptor [55]. In consequence, the entire energy needs to be formed by ATP production via substrate level phosphorylation in glycolysis. Industrial bioethanol fermentations typically start under oxygen presence (aerobic), but permitting further oxygen supply by sealing fermentation vessels after inoculation [56]. With this approach, oxygen depletes due to the anabolic metabolism of the cells [56]. This approach is more gentle than shocking cells by sudden oxygen absence and increases viability of cells at the beginning of the cultivation [57]. Microaerobic conditions are furthermore reported to enhance substrate utilization and ethanol tolerance of *S. cerevisiae* without impairing ethanol yields [55], but at the same time reducing glycerol formation [58]. For these reasons, cultivations in the present study were conducted under microaerobic cultivation conditions in shake flasks that mimic the oxygen situation of large-scale bioreactors.

With regard to l-arabinose uptake, three host-intrinsic transporters with a low-affinity for transporting l-arabinose in *S. cerevisiae* have been reported and comprise the sugar transporters Gal2p, Hxt9p, and Hxt10p [16]. Gal2p is also capable of transporting d-xylose. However, ethanol production upon uptake of d-xylose via Gal2p could not be observed [59]. l-Arabinose transport via the two hexose transporters Hxt9p and Hxt10p were characterized during long-term cultivations of recombinant *S. cerevisiae strains* on l-arabinose [16]. The genes encoding for Hxt9p and Hxt10p were individually overexpressed in a hexose transporter-deficient knockout mutant. Expression of *HXT9* and *HXT10* was found to be repressed by d-glucose [60]. The authors therefore stated that those transporters are hardly expressed under normal fermentation conditions or on sugar mixtures containing d-glucose [16]. *GAL2* expression is reported to be under control of the *GAL2*-promoter which is induced by d-galactose in wild-type *S. cerevisiae* [7, 61, 62].

*GAL2* expression was further reported for being subject to carbon catabolite repression, which prevents the transporter formation in the presence of d-glucose [63, 64]. Next to carbon catabolite repression, d-glucose mediates a mechanism called catabolite inactivation, which caused proteolytical degradation of Gal2p upon d-glucose exposure [63, 64]. *GAL2*, together with the genes of the galactose degradation pathway (Leloir pathway, *GAL1*, *GAL5/PGM2*, *GAL7*, and *GAL10*), belong to the structural *GAL*-genes in *S. cerevisiae*. *GAL1*, *GAL2*, *GAL7*, and *GAL10*, are clustered on two separate chromosomes, each gene with an individual promoter [65]. Regulation of *GAL*-gene expression in *S. cerevisiae* is controlled by the gene products of *GAL3*, *GAL4*, and *GAL80* [65, 66]. *GAL4* codes for a protein that activates transcription of *GAL1*, *GAL2*, *GAL7*, and *GAL10*. The formation of a Gal4p/Gal80p protein complex represses transcription. Repression is relieved by the presence of d-galactose. However, the inducer itself is currently an unknown molecule. *GAL5/PGM2* expression is independent of the other *GAL*-genes and can hence serve as control genes. The impairment in l-arabinose fermentation was partly overcome by overexpression of *GAL2* [67]. Recently, even strain engineering was described where amino acid substitution in *GAL2* evolved strains capable of anaerobic fermentation of l-arabinose in presence of d-glucose and d-xylose by affecting kinetics of l-arabinose and d-glucose transport [68].

Despite it is frequently reported that d-galactose is crucial for *GAL2* expression in wild-type *S. cerevisiae*, the present study demonstrates that *S. cerevisiae* DS61180 is able to induce *GAL2* expression by l-arabinose. Compared to cultivations of the cell lineage on d-glucose, fermentations with l-arabinose as sole carbon source were accompanied by decreased growth rates compared to cultivations on d-glucose and a delayed onset of ethanol production. Comparative transcriptome analyses revealed characteristics of the redox metabolism that indicate a down-regulation of lower glycolysis genes and an up-regulation of redox factor regeneration during growth on l-arabinose, which could serve as potential target for fine tuning the fermentation efficiency of *S. cerevisiae* for the production of lignocellulosic ethanol.

## Methods

### Strains

The industrial *S. cerevisiae* strains DS58227, DS61143, and DS61180 have been developed and provided by DSM (Delft, NL). Strain access is restricted and availability can only be made via DSM. The recombinant strain *S. cerevisiae* DS61143 has been developed from the wild-type *S. cerevisiae* DS58227 by metabolic engineering based on homologous recombination [69–71]. Strain transformation was conducted as described by Gietz and Woods [72]. The strain contains genomically integrated bacterial *araABD* from *Lactobacillus plantarum* and the genes of the non-oxidative PPP are overexpressed [for *TAL1* (transaldolase), *TKL1* (trans-ketolase), *RPE1* (d-ribulose-5-phosphate 3-epimerase), and *RKI1* (Ribose-5-phosphate ketol-isomerase)]. *araABD* are disclosed in [73]. For standard methods for recombinant expression of enzymes in cells, we refer to the handbook of Sambrook and Russel [74]. Additional excessive adaptive evolution steps on l-arabinose of this recombinant strain resulted in *S. cerevisiae* DS61180 [46]. Adaptive evolution was thereby conducted via serial transfer of cultures to arabinose-containing medium based on the readout growth as described by Kuyper et al. [30]. All strains were used for comparative studies about the physiology and transcriptome of the cell lineage during growth on d-glucose. Stain DS61180 was additionally investigated during growth on l-arabinose.

### Media and cultivation conditions

All cultivations were performed at pH 4.2 and 30 °C with minimal medium based on Verduyn [75]. For cultures grown under microaerobic conditions, ergosterol and tween 80 were added to final concentrations of 8 mg/L. d-Glucose, d-galactose, and l-arabinose were used as substrates in a concentration of 20 g/L each for single sugar cultivations and about 10 g/L d-glucose with 10 g/L l-arabinose for mixed sugar cultivations. For cultivations in liquid culture, cells from frozen glycerol stocks were plated on YPD-agar plates (yeast extract peptone glucose) (about 50 h at 30 °C), single colonies were picked and suspended in YPD medium containing 10 g/L yeast extract, 20 g/L peptone, and 20 g/L d-glucose and incubated for about 6 h at 30 °C under aerobic conditions in a rotary shaker (Infors HT Ecotron, Bottmingen, CH) at 110 rpm. YPD cultures were then used to inoculate overnight pre-cultures on minimal medium containing 20 g/L d-glucose. Cells of the aerobic pre-cultures were harvested in the late exponential phase, washed with fresh minimal medium and used to inoculate main cultures to an OD_600nm_ of 0.1. Cultivations were performed under microaerobic conditions using 500-mL unbaffled shake flasks containing 100 mL medium. The shake flasks were equipped with glass water locks and a syringe port for sampling. All cultivations were performed in duplicates.

### Cell disruption, RNA isolation, DNA removal, and reverse transcription

For cell disruption, RNA isolation and the first DNA removal step, the NucleoSpin^®^RNA kit (Macherey–Nagel, Düren, Germany) was used. Cells from shake flask cultivations were harvested in the mid-exponential phase, pelleted and resuspended in 1 mL lysis solution, containing 100 units of lyticase, 2 M sorbitol, and 100 mM EDTA and incubated for 30 min at 30 °C. After centrifugation for 10 min at 13,300 rpm (Heraeus™ Biofuge Fresco™), cell pellets were lysed in 350 µL buffer RA1 and 3.5 µL β-mercaptoethanol by vigorous vortexing for about 7 min and subsequently centrifuged for 5 min at 13,300 rpm. The supernatant was used for further RNA isolation according to the protocol provided by the manufacturer (Macherey–Nagel, Düren, Germany). Potential contaminating DNA in the RNA fraction was removed by digestion with rDNAse according to the NucleoSpin^®^RNA kit (Macherey–Nagel, Düren, Germany). An additional DNA removal step was performed using the Turbo DNA-free™ Kit (Invitrogen™, Life Technologies GmbH, Darmstadt, Germany) according to the protocol. The amounts of isolated RNA and its purity (A260/A230) were determined with a NanoDrop™ 2000 UV–VIS Spectrophotometer (Peqlab, Erlangen, Germany). For the translation of isolated RNA to cDNA the GoScript™ Reverse Transcription System kit (Promega GmbH, Mannheim, Germany) was used with random primers according to the protocol provided by the manufacturer. Resulting cDNA samples were stored at − 20 °C until the measurement by qPCR.

### Primer design for qPCR

The sequences of the genes *GAL2*, *HXT9*, *HXT10* for l-arabinose uptake, *GAL1*, *GAL5/PGM5*, *GAL7*, and *GAL10* from the Leloir pathway, and *SGA1* as endogenous control were blasted via the *Saccharomyces* genome database (http://www.yeastgenome.org/) and specific primers were designed with the software Primer Excess (Life Technologies, Darmstadt, Germany). For primer sequences see Additional file 1: Table S1. *SGA1* encodes a sporulation-specific glucoamylase and was chosen as endogenous control gene [76].

### Quantitative PCR (qPCR) and amplification efficiency

RT-qPCR experiments were performed using the StepOnePlus™ Instrument (Life Technologies, Darmstadt, Germany) in 96-well plates. Two modes were used in this study, the ‘Quantitation-Standard Curve’ mode was used for efficiency determination and the ‘comparative ∆∆*C*_T_’ mode for quantitative comparison. Reactions were carried out from biological duplicates and measured as technical triplicates, each of a quantity of 20 µL per well, containing cDNA samples, forward and reverse primers of the genes of interest and FastStart Universal SYBR Green Master (ROX) (Roche Diagnostics GmbH, Mannheim, Germany) with the fluorophore SYBR Green. The amplification method was set to 65 °C for 1 min for primer annealing with a cycle number of 40. At the end of the run, melting curves analyses were performed at 65 °C to check the amplification quality.

The amplification efficiency was determined for each gene used in RT-qPCR by standard curves prior to the comparative quantification of gene expression levels. For this, dilution series of cDNA were measured with RT-qPCR, the cycle thresholds (*C*_T_ values) were determined for each standard point and plotted against its logarithmic quantity. Each point of the standard curves was measured in triplicates. Efficiency of the standard curves was determined with the software StepOne (Life Technologies, Darmstadt, Germany) by regression.

For raw data evaluation, the comparative ∆∆*c*_T_ method was used. For that purpose, a threshold of 0.4 was set to the amplification plot of each gene of interest (including the endogenous control gene) for all samples. The relative gene expression levels were determined with the software StepOne (Life Technologies, Darmstadt, Germany). For the gene expression levels of *HXT9* and *HXT10*, the cultivation on d-glucose was chosen as reference. For the relative quantification of the expression levels of *GAL*-genes, the cultivation on d-galactose was chosen as reference and as positive control and the cultivation on d-glucose served as negative control for *GAL*-gene expression.

### RNA-sequencing and comparative gene expression analysis

RNA samples were isolated as described before and stored on dry ice for transport. RNA-sequencing was conducted on Illumina NextSeq runs (1 × 75 bp) with 300 M filter reads per run. Samples were analyzed as biological triplicates. Poly(A) library preparation, RNA-sequencing and bioinformatics analysis was performed by Microsynth AG (Balgach, Switzerland). Relevant up- and down-regulated gene expression could be determined by rigorous statistical analysis of genes that were mapped against a reference genome of *S. cerevisiae*. Bioinformatic analysis was performed using standard protocols by applying standard parameters with the following software:

Mapping to the transcriptome was done using the tophat2/bowtie2 framework (tophat2: version 2.1.0 [77–79]; bowtie2: version 2.2.6 [80, 81]). Read counting to features (genes) was done using the HTSeq framework (version 0.6.1.p1 [82]) and statistical analysis was done using DESeq2 from Bioconductor (version 1.8.1 [83]). In order to determine the statistical significance, thresholds for fold change values (FC) as well as corresponding *P*-values were set to |log2FC| ≥ 1 and *P*-value ≤ 0.05. A *P*-value above 0.05 indicates the probability that more than 5% of the reads obtained for the certain genes are false positive and are thus excluded from further considerations. Observable changes in gene expression pattern |log2FC| ≥ 1 × 10^14^ were considered as a significant up- or down-regulation.

### Concentrations of substrates, main- and by-products

The concentrations of d-glucose, d-galactose and l-arabinose, ethanol, glycerol, and acetate were determined by high-performance liquid chromatography using a Hitachi System (VWR Hitachi, High-Technologies Corporation, Tokyo, Japan), equipped with a refractive index (L-2490) detector, an UV–VIS (L-2420) detector and the column 308R-Gel.H (Trentec Analysetechnik, Germany). For analysis, an isocratic method using 5 mM H_2_SO_4_ as eluent with a flow rate of 1 m/min and an oven temperature of 40 °C was used.

### Cell dry weight and correlation with the optical density

Concentration of cell dry weight (g/L) was calculated from optical density values at 600 nm (OD_600_) measured with a spectrophotometer (Libra S11, Biochrome, Cambridge, UK) with a correlation factor of 0.17. The correlation factor was determined for each of the strains used by measuring the weight of dried cell samples from shake flask cultivations, taken over the whole cultivation time, washed with 50 mM ammonium acetate buffer.

## Results

### A metabolic burden caused by strain development is not evident during cultivations on d-glucose as sole carbon source under aerobic conditions in minimal medium

Ethanol production for the industrial strain *S. cerevisiae* DS61180 has already been reported for a substrate mixture of d-glucose, d-galactose, d-xylose, and l-arabinose [84]. However, a precise description of its quantitative physiology during growth on the individual single substrates was missing. Only the comparative analysis of the sugar utilization capacities in these strains allows revealing the metabolic potential of the developed strains. *S. cerevisiae* DS61180 and its two precursor strains, *S. cerevisiae* DS58227 (wild-type) and *S. cerevisiae* DS61143, were thus physiologically characterized on d-glucose as sole carbon and energy source. The cultivations on d-glucose were performed under aerobic cultivation conditions to minimize physiological bias and metabolic limitations in order to achieve unbiased results. The performed experiments aimed at revealing a potential burden (metabolic burden) that resulted from the strain engineering procedures and overexpression of heterologous genes.

All three strains exhibited a comparable growth profile on d-glucose with a characteristic diauxic growth behavior. After a short lag-phase, cells started to grow exponentially for about 10 h. The specific growth rates *µ* of the two recombinant strains, DS61143 and DS61180, exceeded *µ* of the wild-type strain DS58227 by up to 17% (Table 1). Cells entered a second exponential growth phase with decreased specific growth rate (*µ* about 0.1 1/h, see Table 1) upon a period of resting. All cultures reached comparable final biomass titers within 31 h of cultivation.

**Table 1:** Product yields and rates for substrate uptake, growth and product formation during exponential growth of *S. cerevisiae* DS58227, DS61143, and DS61180 in shake flasks under aerobic growth conditions in minimal medium with d-glucose as sole carbon and energy source

Strain	DS58227	DS61143	DS61180
*µ* (h^−1^) (1st exp. phase)	0.30 (± 0.007)	0.36 (± 0.002)	0.33 (± 0.014)
*µ* (h^−1^) (2nd exp. phase)	0.07 (± 0.007)	0.11 (± 0.002)	0.11 (± 0.0002)
*Y*_biomass/glucose_ (g_CDW_/mmol)	0.015 (± 0.001)	0.015 (± 0.001)	0.013 (± 0.001)
*Y*_ethanol/glucose_ (mol/mol)	1.25 (± 0.22)	1.22 (± 0.14)	1.21 (± 0.06)
*Y*_acetate/glucose_ (mol/mol)	0.01 (± 0.02)	0.02 (± 0.004)	0.02 (± 0.01)
*Y*_glycerol/glucose_ (mol/mol)	0.02 (± 0.08)	0.04 (± 0.008)	0.07 (± 0.01)
*r*_glucose_ [mmol/(g_CDW_ h^−1^)]	− 20.61 (± 1.01)	− 24.66 (± 2.16)	− 24.96 (± 0.51)
*r*_ethanol_ [mmol/(g_CDW_ h^−1^)]	25.74 (± 3.20)	29.94 (± 0.84)	30.16 (± 2.09)
*r*_acetate_ [mmol/(g_CDW_ h^−1^)]	0.26 (± 0.36)	0.59 (± 0.15)	0.52 (± 0.35)
*r*_glycerol_ [mmol/(g_CDW_ h^−1^)]	0.46 (± 0.15)	0.94 (± 0.29)	1.78 (± 0.40)

Yields are exclusively given as averages for the first exponential growth phase. Standard deviations are given for two independent measurements

**Fig. 2:**
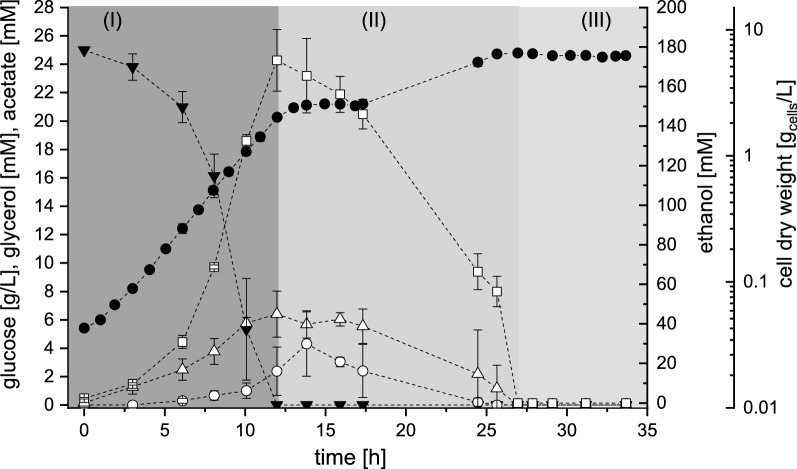
Cultivation profile of *S. cerevisiae* DS61143 in shake flasks under aerobic conditions with d-glucose as sole carbon and energy source. Concentrations over time are given for biomass (filled circle) (cell dry weight), for the substrate d-glucose (filled inverted triangle) and the products ethanol (square), glycerol (triangle), and acetate (square). Error bars indicate standard deviations of two independent measurements. Physiological phases are assigned according to the substrate uptake

Product formation and substrate uptake were comparable for the three strains and is only shown for strain DS61143 (Fig. 2). Glycerol production rates and yields from glucose increased with increasing strain development state. This could be an indication for an impaired redox-metabolism, as it was reported that *S. cerevisiae* uses glycerol production as sink for NADH [52, 85]. Compared to the two recombinant strains, DS58227 showed reduced rates for d-glucose uptake (about 16% lower) and product formation (about 14% for ethanol and up to 55% for acetic acid and glycerol) during exponential growth on d-glucose (Table 1). Strain DS58227 also showed a longer resting phase during the diauxic shift. The strain engineering for obtaining l-arabinose utilization has thus entailed a minor metabolic burden that might point to an increased demand for terminal electron acceptors.

Transcriptome analyses of the three strains supported the observation of a minor metabolic burden, as we found that most genes of the central carbon metabolism were expressed in a comparable manner within the cell lineage. *GAL3*, and genes coding for the glycerol-3 phosphatase Gpp2p and the pyruvate decarboxylase Pdc6p were the only genes whose expression was up-regulated for strain DS61180 compared to DS61143. Remarkable differences in gene expression could be found in PPP genes coding for *RKI1*, *RPE1*, and *TAL1* (Additional file 1: Table S2b). Transcription of those genes was up-regulated in the recombinant strains compared to the wild-type.

### *S. cerevisiae* DS61180 metabolizes l-arabinose as sole carbon and energy source in minimal medium

Next, to elucidate the metabolic potential in terms of ethanol production efficiency of the recombinant l-arabinose pathway, the three strains of the cell lineage were physiologically characterized in microaerobic batch cultivations on l-arabinose as sole carbon and energy source. The cultivations were performed on minimal medium, containing d-glucose as sole substrate for pre-cultures, and l-arabinose as sole substrate for main cultures.

**Fig. 3:**
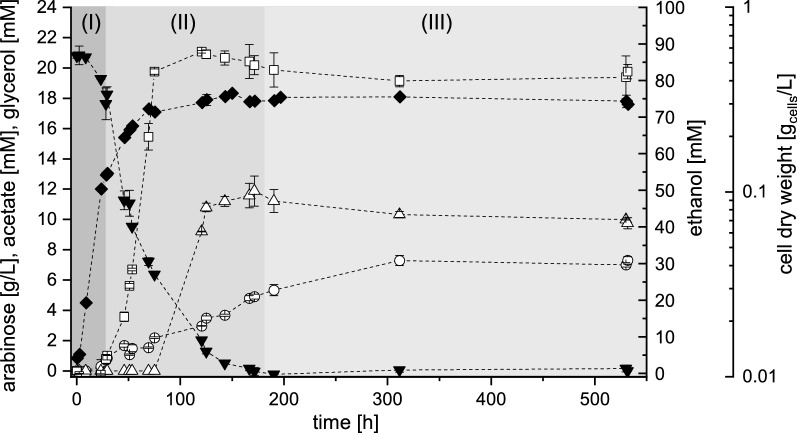
Cultivation profile of *S. cerevisiae* DS61180 in shake flasks under microaerobic conditions with l-arabinose as sole carbon and energy source. Concentrations over time are given for cell dry weight (filled diamond), for l-arabinose (filled inverted triangle) and the products ethanol (square), acetate (circle), and glycerol (triangle). Error bars indicate standard deviations of two independent measurements. Physiological phases are assigned according to the rate of growth and substrate uptake

The strains DS58227 and DS61143 were not able to grow with l-arabinose as sole carbon source, although strain DS61143 theoretically possessed all required genes for l-arabinose conversion. *S. cerevisiae* DS61180 was able to grow with l-arabinose as sole carbon and energy source and its cultivation profile is given in Fig. 3. Growth of strain DS61180 on l-arabinose could be assigned to three phases. Cells grew exponentially for about 26 h, which was significantly slower (phase I, *µ* on l-arabinose: 0.09 1/h) as compared to cultivations on d-glucose. Even though l-arabinose was taken up with a maximal specific uptake rate of 21.26 mmol/(g_cells_ h), DS61180 was not able to convert l-arabinose into ethanol in this phase (phase I). Instead, ethanol production started in the transition phase (phase II) between exponential (phase I) and stationary growth (phase III). This transition phase was also the major production phase for acetate and glycerol. Final ethanol titers of about 80 mM were determined, which corresponded to an overall ethanol yield from l-arabinose of about 0.09 mol/mol. Strain DS61180 was interestingly able to convert l-arabinose to ethanol without demanding galactose for *GAL2*-induction. This is remarkable as d-galactose availability was reported to be mandatory for the formation of Gal2p [7, 65]. Gal2p expression should thus have been impossible without the addition of d-galactose as inducer. This indicates that the adaptive evolutionary engineering on l-arabinose must have caused changes in the strains metabolism that affected l-arabinose uptake and finally enabled strain *S. cerevisiae* DS61180 to grow on l-arabinose as sole carbon source.

### l-Arabinose triggers the induction of *GAL2* expression in *S. cerevisiae* DS61180

Strain DS61180 and DS61143 expressed the heterologous bacterial l-arabinose pathway. However, the cultivations raised the question why strain DS61180 had been capable of utilizing l-arabinose even though l-arabinose utilization was not established for the precursor strain. We hence determined relative gene expression levels of genes coding for the transporters Gal2p, Hxt9p, Hxt10p via RT-qPCR analyses. Insights in transcription of the transporters should clarify if the adaptive evolutionary engineering towards l-arabinose might have enabled strain DS61180 to use Hxt9p or Hxt10p for l-arabinose uptake or to induce *GAL2* expression on l-arabinose in the absence of d-galactose.

Gene expression of *HXT9* and *HXT10* was low in all cases (data not shown). Moreover, expression levels for those transporters were negligible compared to Gal2p. Hence, Hxt9p and Hxt10p could be excluded as cause for the observed l-arabinose utilization of strain DS61180.

**Fig. 4:**
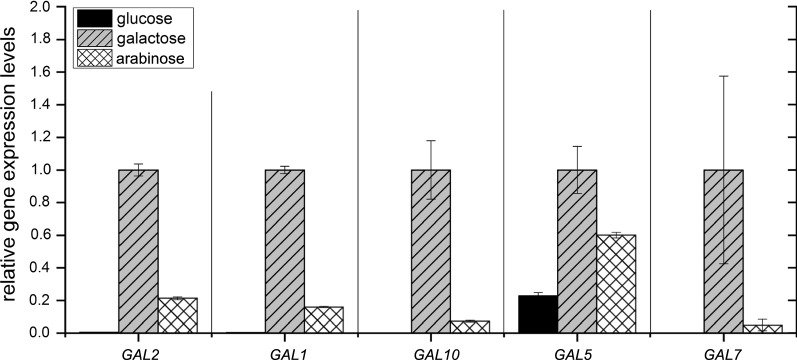
Relative gene expression levels of *GAL2, GAL1, GAL10, GAL5/PGM2*, and *GAL7* during exponential growth of *S. cerevisiae* DS61180 cultivated on d-glucose, d-galactose, or l-arabinose in shake flasks under microaerobic conditions. Relative gene expression levels were determined by the ∆∆*c*_T_ method with the cultivation on d-galactose as reference. Error bars indicate the standard deviation from three measurements of biological duplicates (*n* = 6)

For investigating *GAL2* expression, all cultivations were performed with d-glucose as sole pre-culture substrate as it reportedly represses *GAL2* expression [13]. Due to identical reasons, the main culture on d-glucose served as negative control. The cultivation on d-galactose was used as positive control for *GAL2* expression because of the known inducer capacity of this substrate [66]. As *GAL2* is part of the structural *GAL*-genes that are induced by the same mechanism as transcription of *GAL2*, expression level determination was expanded to the genes *GAL1*, *GAL7*, *GAL5/PGM2*, and *GAL10*. The cultivation on d-galactose has been chosen as reference for data normalization for relative quantification of gene expression levels (Fig. 4).

The highest expression levels of *GAL*-genes were observed during growth on d-galactose (Fig. 4). Interestingly, *GAL*-genes were also expressed during growth on l-arabinose, but at a lower level than on d-galactose. This observation might explain the significantly decreased *µ* of strain DS61180 that was observed during growth on l-arabinose (*µ* = 0.09 1/h) compared to d-galactose (*µ* = 0.36 1/h).

During growth on d-glucose, significant expression among the investigated genes was only visible for *GAL5/PGM2* (Fig. 4). *GAL5/PGM2* expression during growth on d-glucose can be attributed to a d-glucose-independent regulation [65]. In contrast, the remaining investigated *GAL*-genes (*GAL1*, *GAL2*, *GAL7*, and *GAL10*) were regulated in a comparable manner [65]. The observed minor deviation between expression levels of those genes (Fig. 4) can be ascribed to the different promotors of those genes [65].

21% relative expression of *GAL2* during growth on d-galactose could be reached for the cultivation on l-arabinose (Fig. 4, Table 2). It is indicated that l-arabinose itself might had induced *GAL2* expression in the strain DS61180. Quantitative RNA-Seq analysis confirmed this hypothesis, as *GAL*-gene expression was found to be significantly up-regulated during growth of strain DS61180 on l-arabinose compared to growth on d-glucose (Fig. 6, Additional file 1: Table S2a).

**Table 2:** Relative quantity (RQ) of *GAL2* gene expression levels for cultivations of *S. cerevisiae* DS61180 under microaerobic conditions in shake flasks on the substrates d-glucose, d-galactose, and l-arabinose in minimal medium

Substrates	RQ of *GAL2* (%)
Single substrates	
d-Glucose (negative control)	0.52 (± 0.04)
d-Galactose (positive control and reference)	100.01 (± 3.71)
l-Arabinose	21.29 (± 0.84)
Mixed substrates	
d-Glucose/l-arabinose mixture (growth on d-glucose in presence of l-arabinose)	0.23 (± 0.05)
d-Glucose/l-arabinose mixture (growth on l-arabinose after d-glucose depletion)	4.06 (± 0.22)

Substrates were either provided as single substrates or as mixture of d-glucose and l-arabinose. *SGA1* was used as endogenous control and the cultivation on d-galactose served as reference. Given errors indicate standard deviations from three measurements of biological duplicates (*n* = 6)

### l-Arabinose-induced *GAL2* expression is under tight and complex control of d-glucose mediated catabolite repression in *S. cerevisiae* DS61180

**Fig. 5:**
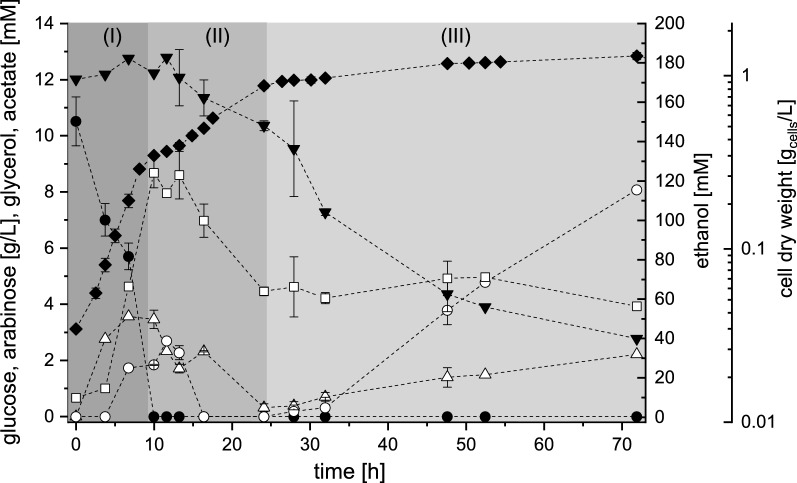
Substrate uptake, product, and biomass formation of *S. cerevisiae* DS61180 in shake flasks under microaerobic conditions grown on d-glucose/l-arabinose substrate mixtures in minimal medium. Concentrations over time are given for cell dry weight (filled diamond), for the substrates d-glucose (filled circle) and l-arabinose (filled inverted triangle), as well as for the metabolites ethanol (square), glycerol (triangle), and acetate (circle). Error bars indicate standard deviations of two independent measurements. Phases are assigned according to the substrate uptake

We demonstrated that l-arabinose is capable of inducing its own uptake via Gal2p in strain DS61180. However, *GAL2* expression is repressed by d-glucose [62]. We therefore investigated growth and product formation on d-glucose/l-arabinose mixtures to evaluate the d-glucose-mediated regulatory mechanism of *GAL2* expression.

d-Glucose and l-arabinose were sequentially consumed by strain DS61180 in mixed sugar cultivations (Fig. 5). d-Glucose was consumed first (phase I). l-Arabinose utilization started upon d-glucose depletion after 10 h of cultivation. Product yields on d-glucose of about 0.22 mol/mol for ethanol, 0.02 mol/mol for glycerol, and 0.003 mol/mol for acetate were reached in phase I. After d-glucose depletion, glycerol, acetate, ethanol, and l-arabinose were simultaneously metabolized (phase II), which is an indication for oxygen absence. The maximal specific l-arabinose uptake rate increased from 1.53 mmol/(g_cells_ h) (phase II) to 3.77 mmol/(g_cells_ h) at the time point when ethanol degradation stopped at concentrations of about 60 mM (beginning of phase III, Fig. 5). The cells produced twofold more acetate but no ethanol during the major l-arabinose consumption phase (phase III).

*GAL2* expression levels determined for the mixed d-glucose/l-arabinose cultivation (Table 2) demonstrate the impairment of l-arabinose utilization by d-glucose (Figs. 4, 5). On the one hand, nearly no gene expression of *GAL2* was visible for samples taken in the mid-exponential growth phase on d-glucose in presence of l-arabinose (growth phase I in Fig. 5). This indicates that catabolite repression of *GAL2* has been active in the presence of d-glucose. On the other hand, about 4% of the *GAL2* expression levels obtained for single sugar cultivations on d-galactose could be reached for growth on l-arabinose (growth phase III in Fig. 5) after d-glucose depletion (Table 2). This means that induction of Gal2p formation had started after d-glucose depletion. However, *GAL2* expression triggered by l-arabinose seems to be less efficient than induction of *GAL2* expression by d-galactose or might be impaired by by-product formation.

Summing up, our results show that strain DS61180 was not able to consume l-arabinose as long as d-glucose was available. This was most likely connected to the repression of *GAL2* expression until d-glucose depleted. Catabolite repression is thus a major determinant of the regulation of l-arabinose uptake in strain DS61180.

### RNA-Seq analysis indicates an increased demand in cofactor regeneration and energy of strain DS61180 during growth on l-arabinose as sole substrate

The question why ethanol production and growth were impaired during the cultivation with l-arabinose as sole carbon and energy source is important for industrial lignocellulosic ethanol production processes. Comparative transcriptome analyses based on RNA-Seq data delivered hints for the cause of these observations. RNA-Seq analysis was performed with strain DS61180 growing exponentially on the single substrates d-glucose and l-arabinose under identical cultivation conditions as used for *GAL*-gene expression analysis by qPCR. Considered genes were limited to the central carbon metabolism. Special attention was paid to genes coding for proteins of the redox, energy and galactose metabolism (*GAL* metabolism). Those genes were supposed to have the highest impact on the metabolism during growth of strain DS61180 on l-arabinose as sole substrate.

**Fig. 6:**
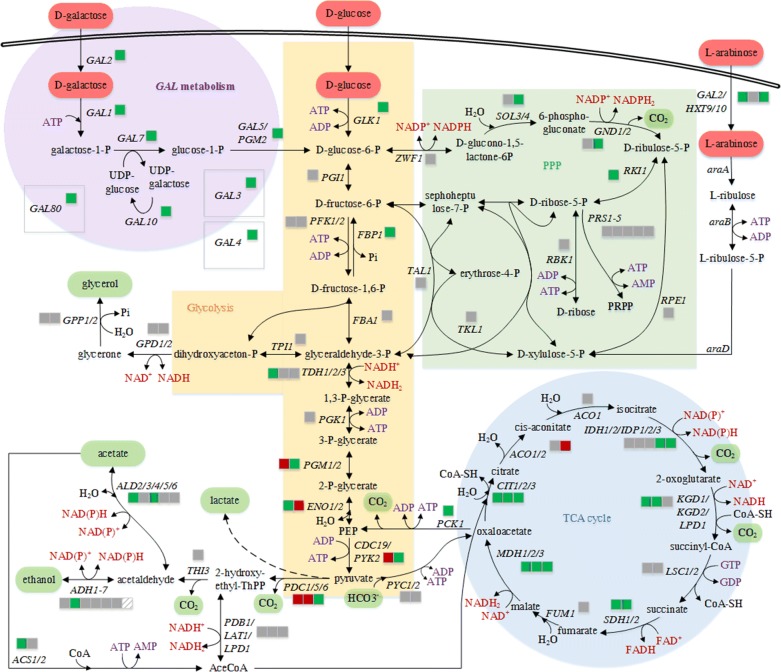
Central carbon metabolism of *S. cerevisiae* including glycolysis, PPP, tricarboxylic acid (TCA) cycle, the *GAL* metabolism, gluconeogenetic reactions and the recombinant arabinose metabolism. Enzyme encoding genes are displayed including regulation according to the comparative transcriptome analysis by RNA-seq. Enzyme names corresponding to the used gene names can be found in the supplemental part (Additional file 1: Table S2). Regulation is indicated by colored boxes close to the gene names. The color code is given as the following: up-regulated (green), down-regulated (red), and not influenced (gray) compared to a chosen reference condition. The boxes display the regulation of strain DS61180 (l-arabinose consuming strain) grown on l-arabinose in relation to its growth on d-glucose as sole substrate. Samples were measured as triplicates and were taken in the mid exponential growth phase during growth on the single substrates

Expression of genes coding for enzymes of the lower glycolysis such as the phosphoglucomutase Pgm1p, the enolase Eno2p, the pyruvate kinases decarboxylases Pdc1p and Pdc5p were down-regulated during growth on l-arabinose (Fig. 6, Additional file 1: Table S2a). Assuming that the down-regulation in transcription of lower glycolysis genes resulted in lowered enzyme concentrations of the respective genes, metabolic fluxes in direction of the TCA cycle and ethanol production were reduced during growth on l-arabinose compared to growth on d-glucose.

Focusing on genes of the redox metabolism, expression of nearly all genes that are involved in the regeneration of the redox factors NAD(P)H was up-regulated during growth on l-arabinose compared to growth on d-glucose (Fig. 6, Additional file 1: Table S2a). This indicates an increased demand for cofactor regeneration when *S. cerevisiae* DS61180 was growing on l-arabinose as sole carbon and energy source. An increased demand in cofactor regeneration might be attributed to the withdrawal of NAD(P)H by the metabolism or for counteracting NAD(P)^+^ accumulation.

Interestingly, significant regulatory differences in metabolic reactions capable of generating ATP could be found for the last reaction of glycolysis and for the gluconeogenetic reaction from d-fructose-1,6-P to d-fructose-6-P catalyzed by FBP1p. The last reaction of glycolysis catalyzes pyruvate synthesis via the pyruvate kinases Pyk2p and Cdc19p. Expression of *CDC19* was down-regulated at the l-arabinose condition, compared to the growth of strain DS61180 on d-glucose (Fig. 6, Additional file 1: Table S2a). In contrast, expression of *FBP1* and *PYK2* is not required for ATP generation during cultivation of *S. cerevisiae* on d-glucose, as their expression is subject to catabolite repression [86, 87]. Hence, the up-regulation of the ATP-generation reactions might hint for an increased demand of energy (ATP) during growth on l-arabinose.

## Discussion

l-Arabinose uptake represents a bottleneck for the efficient fermentation of pentoses with *S. cerevisiae*. It is reported that l-arabinose uptake is the limiting step in the arabinose degradation pathway [16, 35]. However, l-arabinose uptake has been only rarely addressed in studies on l-arabinose utilization [21, 28, 67]. In the present study, l-arabinose was shown to be metabolized by *S. cerevisiae* DS61180 when l-arabinose was provided as sole source of carbon and energy. l-Arabinose transport in our experiments was obviously facilitated via Gal2p, although according to current literature, d-galactose is mandatory for the induction of *GAL2* expression [65]. The tight d-glucose-mediated repression of *GAL2* is further reported to prevent a simultaneous conversion of l-arabinose and d-glucose [36]. In accordance, we found a d-glucose-mediated catabolite repression mechanism when *GAL2* expression has been induced by l-arabinose instead of d-galactose. The activity of this mechanism manifested in sequential utilization of d-glucose and l-arabinose during cultivation on mixed sugars, supported by *GAL2* repression in the d-glucose utilization phase and *GAL2* expression in the l-arabinose utilization phase. The significantly up-regulated *GAL*-gene expression during growth of strain DS61180 on l-arabinose as sole substrate also confirms these findings.

Interestingly, the recombinant precursor strain of *S. cerevisiae* DS61180, DS61143, was not able to use l-arabinose, even though it theoretically possessed all required genes for l-arabinose degradation. The only differences in these two strains must have resulted from adaptive evolutionary engineering on l-arabinose during the strain development procedure. Modifications that enabled strain DS61180 to induce *GAL2* expression in the absence of d-galactose and presence of l-arabinose could thus only had affected the yeast genome or transcriptome. Genome sequencing at DSM (data not shown and access can only be made via DSM) however revealed no mutations [single nucleotide polymorphisms (SNPs)] in the structural *GAL*-genes or in the respective promotor and terminator regions. Special attention was thereby also paid to *GAL3*, *GAL4*, and *GAL80*, which were reported to play a significant regulatory role for *GAL*-gene expression [65]. Binding of Gal80p to the transcriptional activator Gal4p prevents Gal4p from activating *GAL*-gene expression. Synthesis of a currently unidentified inducer molecule is supposed to be catalyzed by Gal3p. This inducer molecule is assumed to prevent Gal80p from binding to *GAL4* upon sensing of intracellular galactose [66]. As SNPs could not be found in the *GAL3*, *GAL4*, and *GAL80* sequences of *S.* *cerevisiae* DS61180, it can be speculated that the modification during the strain development procedure directly affected the inducer and/or the transcriptome of the cells. Although a modification of the inducer could not be experimentally excluded, we found up-regulation of *GAL3*, *GAL4*, and *GAL80* expression during growth of strain DS61180 on l-arabinose. *GAL3* expression was already up-regulated during growth on d-glucose for strain DS61180 compared to DS61143. This makes changes in the inducing mechanism very likely. We therefore assume that the detailed identification of the inducing mechanism, including inducer structure, might be the key for the tuning l-arabinose uptake in *S. cerevisiae*.

One approach to circumvent the bottleneck of d-glucose-mediated repression of l-arabinose uptake could be the implementation of non-*Saccharomyces* l-arabinose transporters in *S. cerevisiae*. Subtil and Boles successfully established and characterized AraTp from the yeast *Scheffersomyces stipitis* and Stp2 from the plant *Arabidopsis thaliana* in *S*. *cerevisiae*. Unfortunately, both transporters were inhibited by d-glucose although they were not supporting the uptake of d-glucose [16]. The Singh group identified two genes coding for l-arabinose transporters of the yeasts *Kluyveromyces marxianus* and *Pichia guilliermondii*, namely *KmAXT1* and *PgAXT1* [88]. Those transporters were capable of transporting d-xylose next to l-arabinose after heterologous expression in *S. cerevisiae*. l-Arabinose uptake by these transporters was also found to be under the regulatory control of d-glucose. As a prominent and more recent example, heterologous expression of a high affinity l-arabinose proton symporter from *P. chrysogenum*, PcAraT, enabled l-arabinose uptake in the presence of d-glucose by *S. cerevisiae* for the first time [89]. Another powerful approach for improving the uptake system for sugar mixtures is to redesign the amino acid sequence of natural *S. cerevisiae* transporters [68, 90]. Variations of only one *GAL2* allele evolved strains with an increased transport activity for l-arabinose and a decreased activity for transporting d-glucose than wild-type Gal2p [68]. In our opinion, a knockout of the d-glucose-mediated repressor or the integration of non-natural l-arabinose transporters in *S. cerevisiae* that are not under the regulatory control of d-glucose possesses a huge potential for mixed sugar cultivations with regard to an industrial ethanol production from lignocellulosic biomass.

Next to the l-arabinose uptake, our results indicate an insufficiency in the metabolic activity of *S. cerevisiae* DS61180 that needs to be compensated for efficient industrial ethanol production from lignocellulosic feedstocks. Our results indicate that l-arabinose had caused a re-direction of pathway activity in order to maintain the balance of cofactors NAD(H) and NADP(H). A lack of NADPH in the metabolism would have promoted an increased demand for cofactor regeneration when cells were growing on l-arabinose. This demand might be so high that under anaerobic conditions the strain is not able to initiate growth on l-arabinose in the absence of oxygen. The oxidative PPP serves as major source for NADPH and no transhydrogenase activity exists in *S. cerevisiae* [27, 91]. The precursors for biomass like nucleic acids cannot be formed without suitable levels of NADPH. Hence, the observed reduction in biomass formation and the transcriptional up-regulation in the oxidative PPP during growth on l-arabinose support this hypothesis. Moreover, ethanol production from acetaldehyde comprises a potential NADPH consuming route during growth of *S. cerevisiae* under microaerobic or anaerobic conditions [52]. We thus assume that the observed decrease in ethanol production from l-arabinose caused a reduced withdrawal of NADPH from the intracellular cofactor-pool. In this case, the decreased ethanol production indicates that an up-regulation of the oxidative PPP is insufficient for keeping the NADPH pool balanced when l-arabinose is supplied as sole carbon and energy source for *S. cerevisiae* DS61180.

A reduced ethanol production potentially originated from a down-regulation of the reaction from acetaldehyde to ethanol, which is catalyzed by a group of NADPH-dependent alcohol dehydrogenases (Adh1-7p) [92]. Alterations in gene expression during growth on l-arabinose were however only visible for *ADH2.* The gene product of *ADH2,* Adh2p, is interestingly mainly responsible for the oxidation of ethanol to acetaldehyde [92]. This excludes alcohol dehydrogenases as origin for the decreased ethanol production during growth on l-arabinose.

Yet, ethanol production is not obligatorily limited by the last catalytic step prior to ethanol formation. A down-regulation of metabolic reactions earlier in the metabolism could also impair ethanol formation. The found transcriptional down-regulation of the lower glycolysis genes (*PGM1*, *ENO2*, *CDC19*, *PDC1*, and *PDC5*) during growth on l-arabinose might had reduced the metabolic flux to the TCA cycle, to acetaldehyde and thus to ethanol. The TCA cycle is important for energy formation under aerobic conditions [28]. The isocitrate dehydrogenase reaction of the TCA cycle, catalyzed by Idp1p, Idp2p, and Idp3p, is a further important source for NADPH [93]. A potential down-regulation of lower glycolysis during initial aerobic growth of the cultivation would have decreased the carbon flux through the TCA cycle, which in turn caused a deficit in both energy generation and NADPH supply. It is possible that the cells were still suffering from this deficit when they switched to fermentative metabolism. About 1 mmol ATP is required per gram biomass and hour for cellular maintenance [94] and 1.67 ATP yields the fermentative metabolism of l-arabinose [95]. Together with the additional withdrawal of ATP by the recombinant bacterial l-arabinose pathway (Fig. 1), we assume that the cells were not capable of compensating this deficit. This could explain both, the low biomass production as well as the impaired ethanol formation during growth on l-arabinose. Another indication for this deficit in ATP was that the strain could not initiate growth on l-arabinose in the absence of oxygen (data not shown). Such detrimental effects on the metabolic state of ethanol-producing yeast were already reported for *S. cerevisiae* that harbored a heterologous d-xylose utilization pathway [96]. Comprehensive and comparative metabolome analyses of cells that were grown on d-xylose as single substrate revealed the up-regulation of the upper glycolysis and PPP genes, down-regulation of the lower glycolysis and a significant increase of the citrate level in the TCA cycle. The authors concluded that these observations resulted in a severely impaired biosynthetic capability and energy balance that lowered ethanol production on xylose compared to glucose [96]. d-Xylose enters the central carbon metabolism of *S. cerevisiae* at the same branch-point as l-arabinose. These findings thus support our hypothesis that bases on physiological characterizations and transcriptomics.

## Conclusion

An efficient industrial bioethanol production necessitates a fast and simultaneous conversion of sugars present in the lignocellulosic biomass hydrolysate, a high ethanol yield, and a low formation rate of by-products. It is therefore important to understand the physiology of the fermenting organisms and the biochemistry behind the conversion of every single sugar present in the feedstock. The isolated consideration of single functional modules of complex systems, like the metabolic routes for single sugars present in the biomass hydrolysate combined with the regulation of interconnected general pathways, delivered individual answers and hints that finally lead to process understanding. In concrete, we found that the d-glucose-mediated regulation of the pentose uptake system omits the efficient and simultaneous fermentation of sugars that are present in the lignocellulose feedstock as it impairs the energy metabolism and in vivo cofactor regeneration. We are thus convinced that pentose uptake systems are required that are independent of the complex regulatory network of d-glucose. Our findings can now serve as basis for engineering and rational process intensification.

## Additional file

**Additional file 1: Table S1.** Specific primer sequences designed for *GAL1*, *GAL2*, *GAL5/PGM2*, *GAL7*, *GAL10*, *HXT9*, *HXT10* and *SGA1* which are used in RT-qPCR. **Table S2.** Comparative RNA-Seq analysis results of genes related to the central carbon metabolism of *S. cerevisiae* DS58227 (wild-type), DS61143 (intermediate strain) and DS61180 (l-arabinose consuming strain). (a) *S. cerevisiae* DS61180 (l-arabinose consuming strain) grown on l-arabinose compared to growth of the same strain on d-glucose as reference. (b) *S. cerevisiae* DS58227 (wild-type) compared to DS61180 (l-arabinose consuming strain) as reference, both grown on d-glucose. (c) *S. cerevisiae* DS61143 (precursor strain of DS61180) compared to DS61180 (l-arabinose consuming strain) as reference, both grown on d-glucose. Genes are assigned to classes that are involved in the redox (I) (genes encoding NAD(P)H dependent proteins), the energy (II) (genes encoding ATP dependent proteins) and the galactose metabolism (III), as well as to a NAD(P)H and ATP independent (IV) class. Samples were analyzed as biological triplicates taken during mid exponential growth of the three strains on the individual substrates d-glucose and/or l-arabinose. Regulation R is indicated as symbols: (**↑**) up-regulated, (-) un-regulated, (**↓**) down-regulated compared to the reference condition that is underlined in the label. E indicates the error of log2FC values.
