# Supplementary material for: l-Arabinose triggers its own uptake via induction of the arabinose-specific Gal2p transporter in an industrial Saccharomyces cerevisiae strain
Source: Biotechnol Biofuels. 2018 Aug 23;11:231. doi: 10.1186/s13068-018-1231-8 (PMC6106821; doi:10.1186/s13068-018-1231-8)
Supplement: Supplementary file 1 — Additional file 1: Table S1. Specific primer sequences designed for GAL1, GAL2, GAL5/PGM2, GAL7, GAL10, HXT9, HXT10 and SGA1 which are used in RT-qPCR. Table S2. Comparative RNA-Seq analysis results of genes related to the central carbon metabolism of S. cerevisiae DS58227 (wild-type), DS61143 (intermediate strain) and DS61180 (l-arabinose consuming strain). (a) S. cerevisiae DS61180 (l-arabinose consuming strain) grown on l-arabinose compared to growth of the same strain on d-glucose as reference. (b) S. cerevisiae DS58227 (wild-type) compared to DS61180 (l-arabinose consuming strain) as reference, both grown on d-glucose. (c) S. cerevisiae DS61143 (precursor strain of DS61180) compared to DS61180 (l-arabinose consuming strain) as reference, both grown on d-glucose. Genes are assigned to classes that are involved in the redox (I) (genes encoding NAD(P)H dependent proteins), the energy (II) (genes encoding ATP dependent proteins) and the galactose metabolism (III), as well as to a NAD(P)H and ATP independent (IV) class. Samples were analyzed as biological triplicates taken during mid exponential growth of the three strains on the individual substrates d-glucose and/or l-arabinose. Regulation R is indicated as symbols: (↑) up-regulated, (-) un-regulated, (↓) down-regulated compared to the reference condition that is underlined in the label. E indicates the error of log2FC values. [file 13068_2018_1231_MOESM1_ESM.docx]

**Additional files**

Table S1: Specific primer sequences designed for GAL1, GAL2 GAL5/PGM2, GAL7, GAL10, HXT9, HXT10 and SGA1 which are used in RT-qPCR.

| Gene | Primer 5`- 3` |
| --- | --- |
| *GAL1* | Fwd: ACTTGCACCGGAAAGGTTTG |
| Galactokinase | Rev: GGTACATCACCCTCACAGAAACTT |
| *GAL2* | Fwd: CCACTGAGAGTCAAGTCGAAATGT |
| Galactose permease | Rev: GCAATCAAGAACCCCCATACC |
| *GAL5/PGM2* | Fwd: ATGTTACCAAATCGGGTGTTGTT |
| Phosphoglucomutase | Rev: CCACAATCGGTGACCTTAAGAGA |
| *GAL7* | Fwd: ATTCCACAAATGAAACAATCAGATCT |
| Galactose-1-phosphate uridylyltransferase | Rev: GGCTTATGATTTTCTCTTGCTTCTCT |
| *GAL10* | Fwd: CGTCGATAGAGAAATTGCTACCTTT |
| UDP-glucose4-epimerase | Rev: ATTTGACTTGGCTTAGCATTTTCA |
| *HXT9* | Fwd: CCAATTACGGTACCAAGAACTATCAC |
| Hexose transporter | Rev: AATGTAGTCCAGGCAAAGCAAAG |
| *HXT10* | Fwd: TGCCTCGATGGCAATTTGT |
| Hexose transporter | Rev: TGCGAAGAAGGTTGGTCTTTC |
| *SGA1* | Fwd: TGGACAAGTCGCTGTCGTATTT |
| Sporulation-specific Glyco Amylase | Rev: TGTCCCGGCGTGTCTGA |

Table S2: Comparative RNA-Seq analysis results of genes related to the central carbon metabolism of *S. cerevisiae* DS58227 (wild-type), DS61143 (intermediate strain) and DS61180 (L-arabinose consuming strain). (a) *S. cerevisiae* DS61180 (L-arabinose consuming strain) grown on L-arabinose compared to growth of the same strain on D-glucose as reference. (b) *S. cerevisiae* DS58227 (wild-type) compared to DS61180 (L-arabinose consuming strain) as reference, both grown on D-glucose. (c) *S. cerevisiae* DS61143 (precursor strain of DS61180) compared to DS61180 (L-arabinose consuming strain) as reference, both grown on D-glucose. Genes are assigned to classes that are involved in the redox (I) (genes encoding NAD(P)H dependent proteins), the energy (II) (genes encoding ATP dependent proteins) and the galactose metabolism (III), as well as to a NAD(P)H and ATP independent (IV) class. Samples were analyzed as biological triplicates taken during mid exponential growth of the three strains on the individual substrates D-glucose and/or L-arabinose. Regulation R is indicated as symbols: (**↑**) up-regulated, (-) un-regulated, (**↓**) down-regulated compared to the reference condition that is underlined in the label. E indicates the error of log2FC values.

|  | |  |  | (a)  DS61180ara vs. DS61180glc | | | | | | (b)  DS58227glc vs. DS61180glc | | | | | | | | (c)  DS61143glc vs.DS61180glc | | | | | | | |
| --- | --- | --- | --- | --- | --- | --- | --- | --- | --- | --- | --- | --- | --- | --- | --- | --- | --- | --- | --- | --- | --- | --- | --- | --- | --- |
| Physiological role | | Gene | Systematic  name | log2FC | | E | p-value | | R | log2FC | | | E | p-value | | R | | log2FC | | | | E | p-value | | R |
| **(I) Redox metabolism** | | | | | | | | | | |  | | | |  |  | |  | | |  | | |  |  |
| 6-phosphogluconate  dehydrogenase | | *GND1* | YHR183W | -0.47 | 0.11 | | na | | **-** | 0.47 | 0.06 | | | | 1.3  *10^-4^ | **-** | | 0.37 | | | 0.05 | | | 0.2 | - |
|  |  | *GND2* | YGR256W | 2.98  *10^14^ | 0.52 | | na | | **↑** | -0.47 | 0.25 | | | | na | **-** | | -0.56 | | | 0.24 | | | na | - |
| glucose-6-P  dehydrogenase | | *ZWF1* | YNL241C | 0.22 | 0.13 | | 0.10 | | **-** | -0.1 | 0.08 | | | | 0.26 | - | | -0.25 | | | 0.06 | | | 2.14  *10^9^ | - |
| alcohol  dehydrogenase | | *ADH1* | YOL086C | -0.25 | 0.1 | | 0.01 | | **-** | 0.46 | 0.06 | | | | 14.2 | - | | 0.26 | | | 0.05 | | | 6.68  *10^5^ | - |
|  |  | *ADH2* | YMR303C | 6.03  *10^14^ | 0.23 | | 2.73  *10^-143^ | | **↑** | -0.03 | 0.25 | | | | 0.9 | - | | 0.05 | | | 0.24 | | | 0.85 | - |
|  |  | *ADH3* | YMR083W | 0.21 | 0.1 | | 0.02 | | **-** | -0.18 | 0.07 | | | | 6.2  *10^-3^ | - | | -0.2 | | | 0.05 | | | 6.74  *10^8^ | - |
|  |  | *ADH4* | YPR004C | -0.09 | 0.07 | | 0.22 | | **-** | 0.18 | 0.06 | | | | 2.8  *10^-3^ | - | | 0.08 | | | 0.05 | | | 0.13 | - |
|  |  | *ADH5* | YBR145W | -0.01 | 0.09 | | 0.91 | | **-** | -0.16 | 0.08 | | | | 0.06 | - | | -0.11 | | | 0.08 | | | 0.15 | - |
|  |  | *ADH6* | YMR318C | -0.34 | 0.09 | | 1.58  *10^-4^ | | **-** | -0.37 | 0.07 | | | | 1.55  *10^6^ | - | | -0.37 | | | 0.06 | | | 9.76  *10^5^ | - |
|  |  | *ADH7* | YCR105W | na | na | | na | | na | na | na | | | | na | na | | na | | | na | | | na | na |
| aldehyde  dehydrogenase | | *ALD2* | YMR170C | 2.27  *10^14^ | 0.11 | | 5.78  *10^-78^ | | **↑** | -0.06 | 0.13 | | | | 0.65 | - | | -0.2 | | | 0.12 | | | 0.84 | - |
|  |  | *ALD3* | YMR169C | -0.35 | 0.72 | | na | | **-** | -0.42 | 0.2 | | | | na | - | | -0.41 | | | 0.2 | | | na | - |
|  |  | *ALD4* | YOR374W | 4.21  *10^11^ | 0.12 | | 4  *10^-235^ | | **↑** | -0.34 | 0.11 | | | | 2.45  *10^-3^ | - | | -0.71 | | | 0.07 | | | 1.82  *10^-11^ | - |
|  |  | *ALD5* | YER073W | -0.56 | 0.1 | | 1.39  *10^6^ | | **-** | -0.02 | 0.07 | | | | 0.81 | - | | -0.09 | | | 0.05 | | | 0.07 | - |
|  |  | *ALD6* | YPL061W | 0.08 | 0.13 | | 0.54 | | **-** | 0.6 | 0.08 | | | | 67.4 | - | | 0.1 | | | 0.06 | | | 0.08 | - |
| glycerol-3-P  dehydrogenase | *GPD1/2* | | YOL059W | 0.33 | 0.12 | | 7.02  *10^-3^ | | **-** | 0.19 | 0.1 | | | | 0.03 | - | | 0,11 | | | 0.07 | | | na | - |
| glyceraldehyde-  3-P dehydrogenase | | *TDH1* | YJL052W | 4.98  *10^14^ | 0.41 | | 4.45  *10^-20^ | | **↑** | 0.44 | 0.25 | | | | na | - | | 0.22 | | | 0.24 | | | 0.35 | - |
|  |  | *TDH2* | YJR009C | -0.66 | 0.11 | | 5.23  *10^5^ | | **-** | 0.61 | 0.07 | | | | 4.94  *10^-6^ | - | | 0.4 | | | 0.05 | | | 2.81  *10^-4^ | - |
|  |  | *TDH3* | YGR192C | -0.61 | 0.11 | | 8.39  *10^6^ | | **-** | 0.29 | 0.07 | | | | na | - | | 0.06 | | | 0.04 | | | na | - |
| isocitrate  dehydrogenase | | *IDH1* | YNL037C | 0.36 | 0.13 | | 4.10  *10^-3^ | | **-** | -0.04 | 0.11 | | | | 0.69 | - | | 0.07 | | | 0.08 | | | 0.41 | - |
|  |  | *IDH2* | YOR136W | 0.50 | 0.08 | | 2.42  *10^5^ | | **-** | -0.06 | 0.08 | | | | na | - | | -0.09 | | | 0.06 | | | 0.17 | - |
|  |  | *IDP1* | YDL066W | -0.53 | 0.09 | | 1.63  *10^5^ | | **-** | 0.05 | 0.06 | | | | 0.44 | - | | 0.1 | | | 0.06 | | | 0.05 | - |
|  |  | *IDP2* | YLR174W | 2.62 *10^14^ | 0.12 | | 1.72  *10^-88^ | | **↑** | -0.28 | 0.14 | | | | 0.04 | - | | -0.31 | | | 0.11 | | | 4.99  *10^-3^ | - |
|  |  | *IDP3* | YNL009W | 2.60  *10^14^ | 0.12 | | 6.97  *10^-92^ | | **↑** | -0.03 | 0.16 | | | | 0.83 | - | | -0.07 | | | 0.17 | | | 0.67 | - |
| alpha-ketoglutarate  dehydrogenase | | *KGD1* | YIL125W | 1.88  *10^14^ | 0.07 | | 1.86  *10^-154^ | | **↑** | -0.05 | 0.07 | | | | 0.46 | - | | -0.14 | | | 0.06 | | | 0.01 | - |
|  |  | *KGD2* | YDR148C | 1.64  *10^14^ | 0.09 | | 1.35  *10^-64^ | | **↑** | -0.17 | 0.08 | | | | 0.03 | - | | -0.27 | | | 0.06 | | | 9.66  *10^8^ | - |
| dihydrolipoamide  dehydrogenase | | *LPD1* | YFL018C | 0.92 | 0.1 | | 9.60  *10^-7^ | | **-** | 0.13 | 0.06 | | | | 0.05 | - | | -0.16 | | | 0.05 | | | 0.77 | - |
| succinate  dehydrogenase | | *SDH1* | YKL148C | 2.58  *10^14^ | 0.11 | | na | | **↑** | -0.02 | 0.1 | | | | 0.82 | - | | -0.2 | | | 0.08 | | | 0.01 | - |
|  |  | *SDH2* | YLL041C | 3.24  *10^14^ | 0.1 | | 0.0 | | **↑** | 0.03 | 0.07 | | | | 0.71 | - | | -0,1 | | | 0,06 | | | 0,12 | - |
| malate  dehydrogenase | | *MDH1* | YKL085W | 1.73  *10^14^ | 0.12 | | 3.98  *10^-32^ | | **↑** | 0.244 | 0.1 | | | | 0.012 | - | | 0.28 | | | 0.08 | | | 3.67  *10^-4^ | - |
|  |  | *MDH2* | YOL126C | 3.20  *10^14^ | 0.11 | | 4.49  *10^-166^ | | **↑** | -0.05 | 0.14 | | | | 0.73 | - | | -0.04 | | | 0.13 | | | 0.78 | - |
|  |  | *MDH3* | YDL078C | 1.82  *10^14^ | 0.1 | | 5.30  *10^-69^ | | **↑** | -0.19 | 0.07 | | | | 6.0*10^-3^ | - | | -0.24 | | | 0.06 | | | 1.26  *10^-4^ | - |
| **(II) Energy metabolism** | | | | | | | | | | |  | | | |  |  | |  | | |  | | |  |  |
| glucokinase | | *GLK1* | YCL040W | 3.69  *10^14^ | 0.17 | | 1.93  *10^-90^ | | ↑ | -0.19 | 0.2 | | | | 0.36 | - | | -0.63 | | | 0.18 | | | 6.47  *10^-4^ | - |
| phosphofructokinase | | *PFK2* | YMR205C | -0.60 | 0.7 | | na | | - | 0.26 | 0.07 | | | | 9.34  *10^9^ | - | | 0.17 | | | 0.05 | | | 8.54  *10^-4^ | - |
|  |  | *PFK1* | YGR240C | -0.34 | 0.53 | | 0.52 | | - | 0.41 | 0.65 | | | | 2.84  *10^4^ | - | | 0.29 | | | 0.05 | | | 1.61  *10^6^ | - |
| fructose-1,6-bisphosphatase | | *FBP1* | YLR377C | 6.30  *10^14^ | 0.18 | | 2.01  *10^-249^ | | ↑ | 0.5 | 0.22 | | | | 0.03 | - | | 0.03 | | | 0.23 | | | 0.88 | - |
| pyruvate kinase | | *CDC19* | YAL038W | 1.1  *10^14^ | 0.13 | | 1.07  *10^-3^ | | ↓ | 0.22 | 0.07 | | | | 7.69  *10^-4^ | - | | 0.11 | | | 0.05 | | | 0.04 | - |
|  |  | *PYK2* | YOR347C | 1.79  *10^14^ | 0.11 | | 7.93  *10^-45^ | | ↑ | -0.14 | 0.1 | | | | 0.17 | - | | -0.3 | | | 0.1 | | | 1.69  *10^-3^ | - |
| 3-phosphoglycerate  kinase | | *PGK1* | YCR012W | 0.16 | 0.11 | | 0.13 | | - | 0.45 | 0.07 | | | | 1.1  *10^4^ | - | | 0.26 | | | 0.06 | | | 4.94  *10^8^ | - |
| galactokinase | | *GAL1* | YBR020W | 9.58  *10^13^ | 0.12 | | 0.0 | | ↑ | 0.26 | 0.2 | | | | 0.19 | - | | 0.13 | | | 0.2 | | | 0.53 | - |
| phosphoenolpyruvate  carboxykinase | | *PCK1* | YKR097W | 6.77  *10^14^ | 0.09 | | 0.0 | | ↑ | -0.39 | 0.14 | | | | 6.12  *10^-3^ | - | | -0.48 | | | 0.14 | | | 7.05  *10^-4^ | - |
| phosphoribosylpyrophosphate  synthetase | | *PRS1* | YKL181W | -0.64 | 0.09 | | 5.43 | | - | -0.05 | 0.06 | | | | 0.37 | - | | -0.07 | | | 0.05 | | | 0.2 | - |
|  |  | *PRS2* | YER099C | 0.51 | 0.1 | | 3.22  *10^6^ | | - | 0.11 | 0.08 | | | | 0.16 | - | | 0.22 | | | 0.07 | | | 2.83  *10^-3^ | - |
|  |  | *PRS3* | YHL011C | 0.05 | 0.07 | | 0.46 | | - | 0.02 | 0.07 | | | | 0.74 | - | | -0.5 | | | 0.06 | | | 0.46 | - |
|  |  | *PRS4* | YBL068W | -0.33 | 0.1 | | 1.07  *10^-3^ | | - | 0.02 | 0.1 | | | | 0.85 | - | | 0.02 | | | 0.08 | | | 0.81 | - |
|  |  | *PRS5* | YOL061W | -0.70 | 0.1 | | 0.20 | | - | -0.14 | 0.06 | | | | 0.03 | - | | -0.2 | | | 0.06 | | | 4.7  *10^-4^ | - |
| pyruvate  carboxylase | | *PYC1* | YGL062W | 0.60 | 0.08 | | na | | - | 0.17 | 0.08 | | | | 0.03 | - | | -0.02 | | | 0.05 | | | 0.62 | - |
|  |  | *PYC2* | YBR218C | 0.10 | 0.08 | | 0.31 | | - | -0.16 | 0.08 | | | | 0.05 | - | | -0.15 | | | 0.06 | | | 7.12  *10^-3^ | - |
| ribokinase | | *RBK1* | YCR036W | 0.66 | 0.08 | | 2.97  *10^-3^ | | - | 0.02 | 0.07 | | | | 0.77 | - | | 0.03 | | | 0.07 | | | 0.7 | - |
| acetyl-coA  synthetase | | *ACS1* | YAL054C | 4.60  *10^14^ | 0.12 | | 0.0 | | ↑ | -0.23 | 0.14 | | | | 0.1 | - | | -0.45 | | | 0.15 | | | 3.21  *10^-3^ | - |
|  |  | *ACS2* | YLR153C | -0.84 | 0.11 | | 5.01 | | - | 0.06 | 0.07 | | | | 0.39 | - | | 0.01 | | | 0.06 | | | 0.87 | - |
| ligase of  succinyl-CoA | | *LSC1* | YOR142W | -0.17 | 0.09 | | 0.05 | | - | na | na | | | | na | na | | na | | | na | | | na | na |
|  |  | *LSC2* | YGR244C | 0.74 | 0.1 | | 0.01 | | - | 0.21 | 0.08 | | | | 0.01 | - | | 0.15 | | | 0.06 | | | 0.02 | - |
| **(III) Galactose metabolism** | | | | | | | | | | | | | | | | | | | | | | | | | |
| galactokinase | | *GAL1* | YBR020W | 9.5  *10^13^ | 0.12 | | 0.0 | ↑ | | 0.26 | 0.2 | | | | 0.19 | - | | | 0.13 | 0.2 | | | | 0.53 | - |
| galactose permease | | *GAL2* | YLR081W | 1.14  *10^14^ | 0.18 | | 0.0 | ↑ | | -0.46 | 0.25 | | | | 0.06 | - | | | -0.34 | 0.24 | | | | 0.16 | - |
| transcriptional  regulator | | *GAL3* | YDR009W | 4.08  *10^14^ | 0.11 | | 0.0 | ↑ | | -0.89 | 0.18 | | | | 3.56  *10^7^ | - | | | -1.22  *10^14^ | 0.18 | | | | 419 | ↓ |
| **galactose-responsive** transcription factor | | *GAL4* | YPL248C | 2.95  *10^14^ | 0.15 | | 7.90  *10^-76^ | ↑ | | 0.13 | 0.2 | | | | 0.51 | - | | | 0.18 | 0.2 | | | | 0.34 | - |
| phosphoglucomutase | | *GAL5/PGM2* | YMR105C | 3.40  *10^14^ | 0.34 | | 2.45  *10^-9^ | ↑ | | -0.08 | 0.24 | | | | 0.75 | - | | | -0.25 | 0.23 | | | | na | - |
| galactose-1-P uridyl transferase | | *GAL7* | YBR018C | 9.76  *10^14^ | 0.39 | | 1.13  *10^-124^ | ↑ | | -0.14 | 0.45 | | | | 0.6 | - | | | -0.25 | 0.24 | | | | 0.3 | - |
| UDP-glucose-4-epimerase | | *GAL10* | YBR019C | 1.02  *10^14^ | 0.16 | | 0.0 | ↑ | | -0.28 | 0.23 | | | | 0.24 | - | | | 0.1 | 0.23 | | | | 0.66 | - |
| transcriptional  regulator | | *GAL80* | YML051W | 1.09  *10^14^ | 0.10 | | 8.43  *10^-24^ | ↑ | | -0.23 | 0.08 | | | | 5.59  *10^-3^ | - | | | -0.25 | 0.09 | | | | 6.16  *10^-3^ | - |
| **(IV) NAD(P)H/ATP independent genes** | | | | | | | | | | | | | | | | | | | | | | | | |  |
| glycerol-3 phosphatase | | *GPP1* | YIL053W | -0.52 | 0.16 | | 1.26  *10^-3^ | | **-** | 0.15 | | 0.1 | | | 0.1 | **-** | | -0.03 | | 0.09 | | | | 0.76 | - |
|  |  | *GPP2* | YER062C | 0.42 | 0.16 | | 7.26  *10^-3^ | | **-** | -0.51 | | 0.11 | | | 6.45  *10^8^ | **-** | | -1  *10^14^ | | 0.10 | | | | 1.08  *10^-8^ | **↓** |
| pyruvate  decarboxylase | | *PDC1* | YLR044C | -1.14  *10^14^ | 0.13 | | 3.32  *10^-5^ | | **↓** | 0.44 | | 0.07 | | | 3.03  *10^4^ | **-** | | 0.33 | | 0.06 | | | | 3.84  *10^5^ | - |
|  |  | *PDC5* | YLR134W | -1.06  *10^14^ | 0.15 | | 142 | | **↓** | 0.29 | | 0.13 | | | 0.03 | **-** | | 0.37 | | 0.12 | | | | 1.77  *10^-3^ | - |
|  |  | *PDC6* | YGR087C | 2.02  *10^14^ | 0.15 | | 1.51  *10^-29^ | | **↑** | -2  *10^14^ | | 0.14 | | | 6.62  *10^-21^ | **↓** | | -2  *10^14^ | | 0.14 | | | | 1.46  *10^-28^ | **↓** |
| fructose 1,6-bisphosphate  aldolase | | *FBA1* | YKL060C | -0.44 | 0.11 | | 9.98  *10^9^ | | **-** | 0.39 | | 0.07 | | | 9.32  *10^5^ | **-** | | 0.28 | | 0.05 | | | | 7.67*10^6^ | - |
| triose-phosphate  isomerase | | *TPI1* | YDR050C | -0.4 | 0.1 | | 4,58  *10^9^ | | **-** | 0.48 | | 0.06 | | | 0.87 | **-** | | 0.39 | | 0.05 | | | | 0.43 | - |
| phosphogluco-isomerase | | *PGI1* | YBR196C | 0.51 | 0.60 | | 0.4 | | **-** | 0.24 | | 0.06 | | | 01.51  *10^-4^ | **-** | | 0.1 | | 0.23 | | | | 0.65 | - |
| enolase | | *ENO1* | YGR254W | 2.34  *10^14^ | 0.12 | | 1.89  *10^-66^ | | **↑** | 1  *10^14^ | | 0.16 | | | 2.99  *10^3^ | **↑** | | 0.71 | | 0.07 | | | | 7.98  *10^-9^ | - |
|  |  | *ENO2* | YHR174W | -1.26  *10^14^ | 0.12 | | 9.02  *10^-11^ | | **↓** | 0.3 | | 0.07 | | | 3.02  *10^9^ | **-** | | 0.2 | | 0.05 | | | | 1.6  *10^-4^ | - |
| thiamine  metabolism | | *THI3* | YDL080C | -0.12 | 0.1 | | 0.17 | | **-** | -8.1  *10^-3^ | | 0.08 | | | 0.92 | **-** | | 0.06 | | 0.07 | | | | 0.4 | - |
| transaldolase | | *TAL1* | YLR354C | -0.6 | 0.08 | | 0.85 | | **-** | -2  *10^14^ | | 0.06 | | | 1.52  *10^-175^ | **↓** | | 0.33 | | 0.05 | | | | 1.2  *10^3^ | - |
| 6-P-gluconolactonase | | *SOL3* | YHR163W | -0.52 | 0.1 | | 2.33  *10^6^ | | **-** | 0.35 | | 0.06 | | | 3.07  *10^4^ | **-** | | 0.31 | | 0.06 | | | | 3.76  *10^6^ | - |
|  |  | *SOL4* | YGR248W | 1.25  *10^13^ | 0.71 | | 0.1 | | **↑** | -0.41 | | 0.21 | | | 0.05 | **-** | | -0.48 | | 0.2 | | | | 0.02 | - |
| phosphoglucomutase | | *PGM1* | YKL127W | -1.38  *10^14^ | 0.08 | | 2.27  *10^-49^ | | **↓** | 0.08 | | 0.07 | | | 0.28 | **-** | | 0.06 | | 0.06 | | | | 0.32 | - |
| ribulose-5-P epimerase | | *RPE1* | YJL121C | -0.12 | 0.12 | | 0.32 | | **-** | -3  *10^14^ | | 0.07 | | | 0 | **↓** | | -0.1 | | 0.06 | | | | 0.11 | - |
| transketolase | | *TKL1* | YPR074C | 01.11*10^-3^ | 0.10 | | 1 | | **-** | 0.03 | | 0.07 | | | 0.6 | **-** | | 0.23 | | 0.06 | | | | 4.3  *10^9^ | - |
| ribose-5-P  ketol-isomerase | | *RKI1* | YOR095C | 1.02  *10^14^ | 0.08 | | 2.26  *10^-28^ | | **↑** | -3  *10^14^ | | 0.08 | | | 1.64  *10^-225^ | | **↓** | 0.8 | | 0.06 | | | | 7.36  *10^-3^ | - |
| hexose transporter | | *HXT9* | YJL219W | -0.14 | 0.42 | | 0.75 | | **-** | -0.11 | | 0.25 | | | 0.66 | **-** | | -0.45 | | 0.24 | | | | 0.06 | - |
|  |  | *HXT10* | YFL011W | 3.18  *10^14^ | 0.35 | | 1.49  *10^-5^ | | **↑** | 9  *10^-3^ | | 0.22 | | | 0.97 | **-** | | -0.04 | | 0.21 | | | | 0.85 | - |
| aconitase | | *ACO1* | YLR304C | 0.44 | 0.08 | | 5.67  *10^5^ | | **-** | 0.09 | | 0.08 | | | 0.28 | **-** | | 0.07 | | 0.05 | | | | 0.19 | - |
|  |  | *ACO2* | YJL200C | -1.7  *10^14^ | 0.08 | | 7.42  *10^-95^ | | **↓** | 0.21 | | 0.06 | | | 3.77  *10^-4^ | **-** | | 0.27 | | 0.05 | | | | 2.05  *10^7^ | - |
| fumarase | | *FUM1* | YPL262W | 0.51 | 0.1 | | 6.85  *10^5^ | | **-** | 0.03 | | 0.07 | | | 0.66 | **-** | | -0.11 | | 0.06 | | | | 0.04 | - |
| citrate synthase | | *CIT1* | YNR001C | 2.22  *10^14^ | 0.1 | | 9.02  *10^-92^ | | **↑** | -0.2 | | 0,11 | | | 0,07 | **-** | | -0.45 | | 0.08 | | | | 1.45  *10^6^ | - |
|  |  | *CIT2* | YCR005C | 1.3  *10^14^ | 0.1 | | 2.25  *10^-27^ | | **↑** | -0,82 | | 0,07 | | | 1-59  *10^-14^ | **-** | | -0.52 | | 0.08 | | | | 1.24  *10^3^ | - |
|  |  | *CIT3* | YPR001W | 3.8  *10^14^ | 0.1 | | 2.28  *10^-283^ | | **↑** | -0.49 | | 0.17 | | | 4.22  *10^-3^ | **-** | | -0.49 | | 0.17 | | | | 3.7  *10^-3^ | - |
